# Climatic Influence on the Morals

**Published:** 1888-10

**Authors:** Felix L. Oswald


### Selections.

### *CLIMATIC INFLUENCE ON THE MORALS.*

Modern French scientists are nothing if not methodical, and have repeatedly called attention to the curious regularity in the geographical distribution of certain vices and virtues: intemperance, for instance, north of the forty-eighth parallel; sexual aberrations, south of the forty-fifth; financial extravagance, in large seaport towns; thrift, in pastoral highland regions. It is, indeed, a remarkable circumstance, that in the home of the best wine-grapes, in Greece and southern Spain, drunkenness is far less prevalent than in Scotland, or in Russian Poland, where Bacchus can tempt his votaries only with nauseous vodka. The idea that a low temperature begets an instinctive craving for alcoholic tonics seems disproved by the teetotalism of the Patagonian savages, who horsewhip every Spanish stimulant-monger without benefit of clergy. The Lesghian mountaineers, too, observe the interdict of the Koran in the icy summit regions of the Caucasus; but there is no doubt that the bracing influence of a cold climate affords a certain degree of immunity from the debilitating effect of the alcohol vice, and that the Scandinavian peasant can for years survive the effects of a daily dose of alcohol that would kill an Egyptian fellah in a single month. But it is equally certain that the temperance of Southland nations is considerably facilitated by the abundance of non-alcoholic pastimes. The Spaniards have their fandangos and bull-fights, the Greeks their border raids, cocking-mains and horse races; while the Scotchman, after six days of hard work, is confronted with the choice between the delirium of an alcohol fever and the appalling tedium of Sabbatarian asceticism, and naturally chooses the less dismal alternative.

The question, though, remains if religious gloom itself is not an outcome of climatic influences. Cardinal de Retz, indeed, held that orthodox loyalty is a flower that cannot flourish north of the Alps; but it is more than probable that the survival of that plant has been greatly assisted by the conniving *bonhomie* of South European ecclesiastics, who, centuries ago, began to appreciate the wisdom of extending the practice of renunciation to the claim of consistency.—*Dr. Felix L. Oswald, in Popular Science Monthly.*

